# Phlegmonous Gastritis During Consolidation Therapy With High-Dose Cytarabine and FLT3 Inhibitor in FLT3-ITD-Mutated Acute Myeloid Leukemia

**DOI:** 10.7759/cureus.113583

**Published:** 2026-07-29

**Authors:** Hidekazu Itamura, Tatsuya Mihashi, Yusuke Hirano, Shinya Kimura

**Affiliations:** 1 Division of Hematology, Respiratory Medicine and Oncology, Department of Internal Medicine, Faculty of Medicine, Saga University, Saga, JPN; 2 Department of Transfusion, Saga University Hospital, Saga, JPN

**Keywords:** acute myeloid leukemia (aml), acute phlegmonous gastritis, enterococcus faecium, flt3-inhibitor, vonoprazan

## Abstract

Phlegmonous gastritis (PG) is a rare disorder in which a bacterial infection arises in the gastric wall. We report a patient in his 60s with FMS-like tyrosine kinase 3 internal tandem duplication (FLT3-ITD)-mutated acute myeloid leukemia (AML) who developed PG during the consolidation therapy with high-dose cytarabine and the FLT3 inhibitor quizartinib. The patient developed fever, abdominal pain, hematemesis, and hypovolemic shock during neutropenia on day 17. Contrast-enhanced computed tomography (CT) showed edematous thickening of the gastric wall with perigastric fat stranding. Upper gastrointestinal endoscopy revealed diffuse edematous mucosa with erosions, and a gastric biopsy culture yielded *Enterococcus faecium*. Intravenous piperacillin/tazobactam, followed by ampicillin/sulbactam after clinical improvement, achieved complete clinical and radiologic resolution without surgical intervention. This case highlights the importance of early suspicion, prompt contrast-enhanced CT, and endoscopic biopsy with concurrent microbiological evaluation, followed by timely broad-spectrum antimicrobial therapy in immunocompromised patients with chemotherapy-induced neutropenia in the setting of hematological malignancy.

## Introduction

Acute phlegmonous gastritis (PG) is a rare but potentially fatal bacterial infection of the gastric wall, characterized by acute suppurative inflammation predominantly involving the submucosa and occasionally extending to the muscularis propria [1,2]. Because clinical manifestations, including epigastric pain, fever, nausea, and vomiting, are nonspecific, early diagnosis can be challenging. However, delayed recognition may lead to septic shock, gastric necrosis, perforation, or death [3]. Contrast-enhanced computed tomography (CT) is useful for early detection, and prompt administration of broad-spectrum intravenous antibiotics has enabled successful nonoperative management in selected patients [4,5]. Although streptococci, particularly *Streptococcus pyogenes*, are traditionally regarded as the most common causative organisms, multiple other bacterial pathogens have been implicated [2,6,7]. Proposed mechanisms include direct invasion through damaged gastric mucosa, contiguous spread from adjacent infected organs, and hematogenous dissemination.

PG associated with hematologic malignancies is extremely uncommon. Previous reports have described PG during chemotherapy-induced neutropenia in induction therapy, immunosuppressants use, and, rarely, as an initial manifestation of acute myeloid leukemia (AML) [6-8]. Here, we describe a first reported case of PG caused by *Enterococcus faecium* during consolidation therapy with the FLT3 inhibitor quizartinib in a patient with FMS-like tyrosine kinase 3 internal tandem duplication (FLT3-ITD)-mutated AML, successfully managed with antimicrobial therapy alone. Because the symptoms of PG are nonspecific and may be atypical or muted in neutropenic patients, early clinical suspicion and prompt contrast-enhanced CT are particularly important for timely diagnosis.

## Case presentation

**Figure 1:**
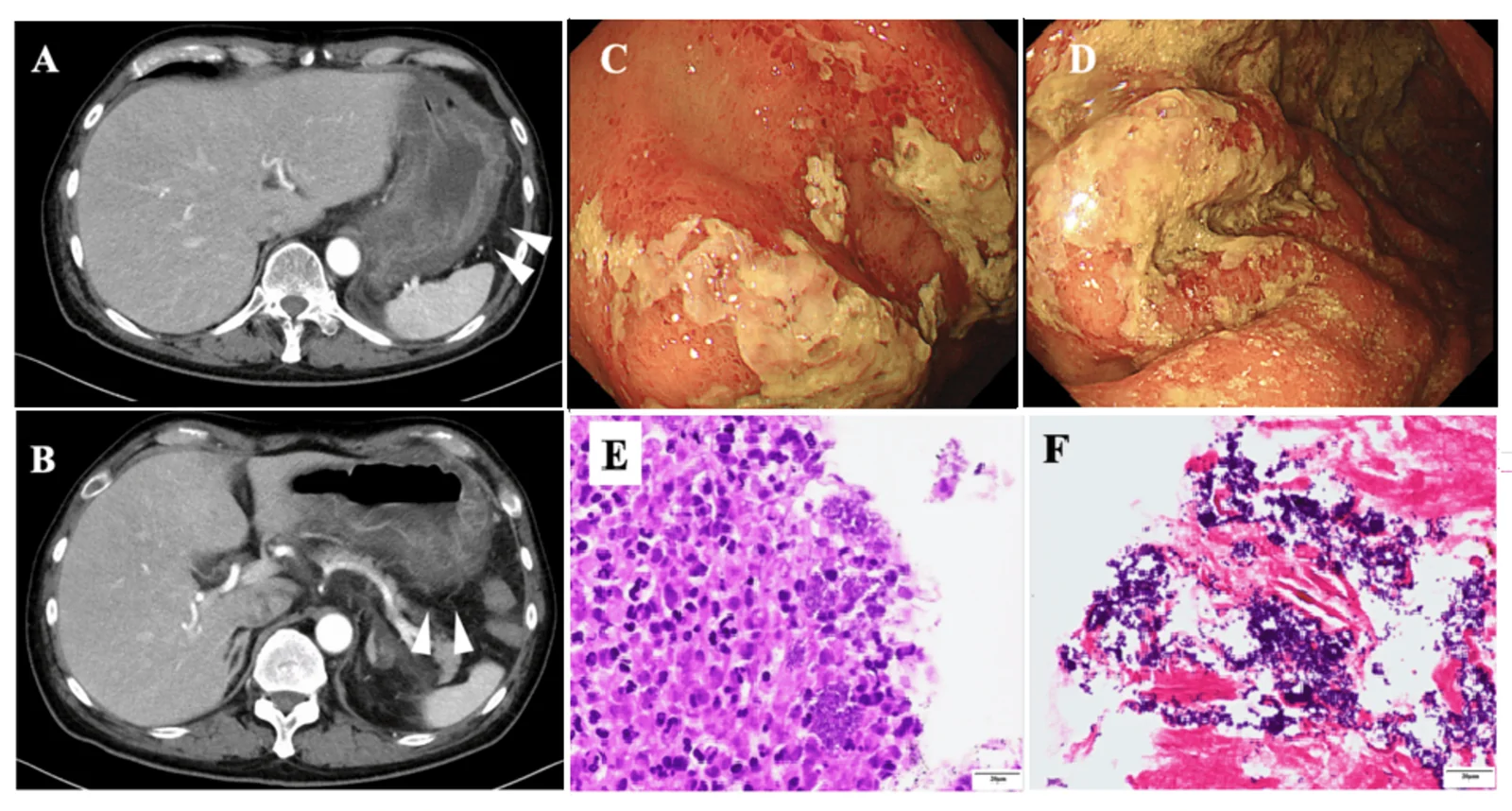
Diagnostic imaging, endoscopic, and histological findings in PG. (A, B) Contrast-enhanced computed tomography on day 18 showing diffuse gastric wall thickening and perigastric fat stranding (arrows). (C, D) Upper gastrointestinal endoscopy on day 21 showing diffusely edematous mucosa with erythema and erosions in the gastric body. (E, F) Histopathologic findings of the gastric biopsy specimen. (E) Hematoxylin and eosin staining showing marked neutrophilic inflammation with necrotic inflammatory exudate in the gastric mucosa (original magnification ×400). (F) Gram staining showing Gram-positive cocci within the inflammatory exudate and gastric mucosal tissue (original magnification ×400). CT: computed tomography; PG: phlegmonous gastritis.

A Japanese man in his 60s with AML harboring an FLT3-ITD mutation achieved complete remission after initial induction chemotherapy with idarubicin and cytarabine. As post-remission therapy, high-dose cytarabine (HiDAC; 1.5 g/m^2^ intravenously every 12 hours on days 1, 3, and 5) in combination with the FLT3 inhibitor quizartinib was administered (17.7 mg orally once daily on days 6-17). There was no anti-bacterial prophylaxis. He had also received vonoprazan fumarate for gastroesophageal reflux disease from induction therapy. During the third course of HiDAC plus quizartinib, the patient developed chemotherapy-induced bone marrow suppression with severe neutropenia. While granulocyte colony-stimulating factor was initiated from day 8, the absolute neutrophil count (ANC) fell below 0.5 × 10^9^/L on day 12. On day 17, the patient suddenly developed chills, fever, and vomiting containing blood clots and fresh blood. The white blood cell count and ANC were 0.29 × 10^9^/L and 0.05 × 10^9^/L, respectively. Empiric intravenous cefepime was initiated the same day for febrile neutropenia. Quizartinib was discontinued on day 17. The patient continued to have fever that persisted above 38°C into the following day, developed shock (blood pressure, 72/38 mmHg), and was transferred to the intensive care unit on day 18. Contrast-enhanced CT demonstrated edematous thickening of the gastric wall with prominent perigastric fat stranding extending into the left abdominal cavity (Figure 1A, B). No other obvious source of infection was detected on CT.

Based on the CT findings, a gastrointestinal infection was suspected despite ongoing cefepime therapy. The antimicrobial regimen was therefore switched to piperacillin/tazobactam to provide broader-spectrum coverage, including anaerobic organisms from day 18 to day 21. After that, vital signs stabilized rapidly, and epigastric pain, which had been most severe on day 17, gradually improved. An extensive diagnostic work-up for the infectious focus, including blood cultures from peripheral blood and the central venous catheter, urine, and sputum cultures, failed to yield any significant pathogen. *Clostridioides difficile *toxin assay was also negative. The central venous catheter insertion site showed no erythema, tenderness, or discharge, and catheter-related infection was considered unlikely.

On day 21, an upper gastrointestinal endoscopy revealed diffusely edematous gastric mucosa (Figure 1C, D). From the cardia to the angular incisure, multiple areas of erythema and superficial erosions were observed. Relatively large erosions were noted on the posterior wall of the angulus and along the greater curvature of the upper gastric body. Yellow-brown debris was present within the gastric lumen and was partly adherent to the margins of these erosions, raising suspicion of necrotic material.

Targeted biopsies were obtained from the erosions on the posterior wall of the angulus. Histologically, no overt neoplastic lesion was identified. Inflammatory cell infiltration, predominantly composed of neutrophils and lymphocytes, was observed. The inflammatory exudate and gastric mucosal tissue contained numerous neutrophils and aggregates of Gram-positive bacteria (Figure 1E, F). Grocott methenamine silver staining showed only rare spore-like structures without evidence of fungal hyphae, effectively excluding invasive fungal infection. Immunohistochemical staining for cytomegalovirus showed no significant findings. Bacterial culture of the gastric specimens grew *Enterococcus faecium*. Taken together, the clinical course during the neutropenic phase, radiologic findings, and endoscopic, histopathologic, and microbiologic results supported the diagnosis of PG.

**Figure 2:**
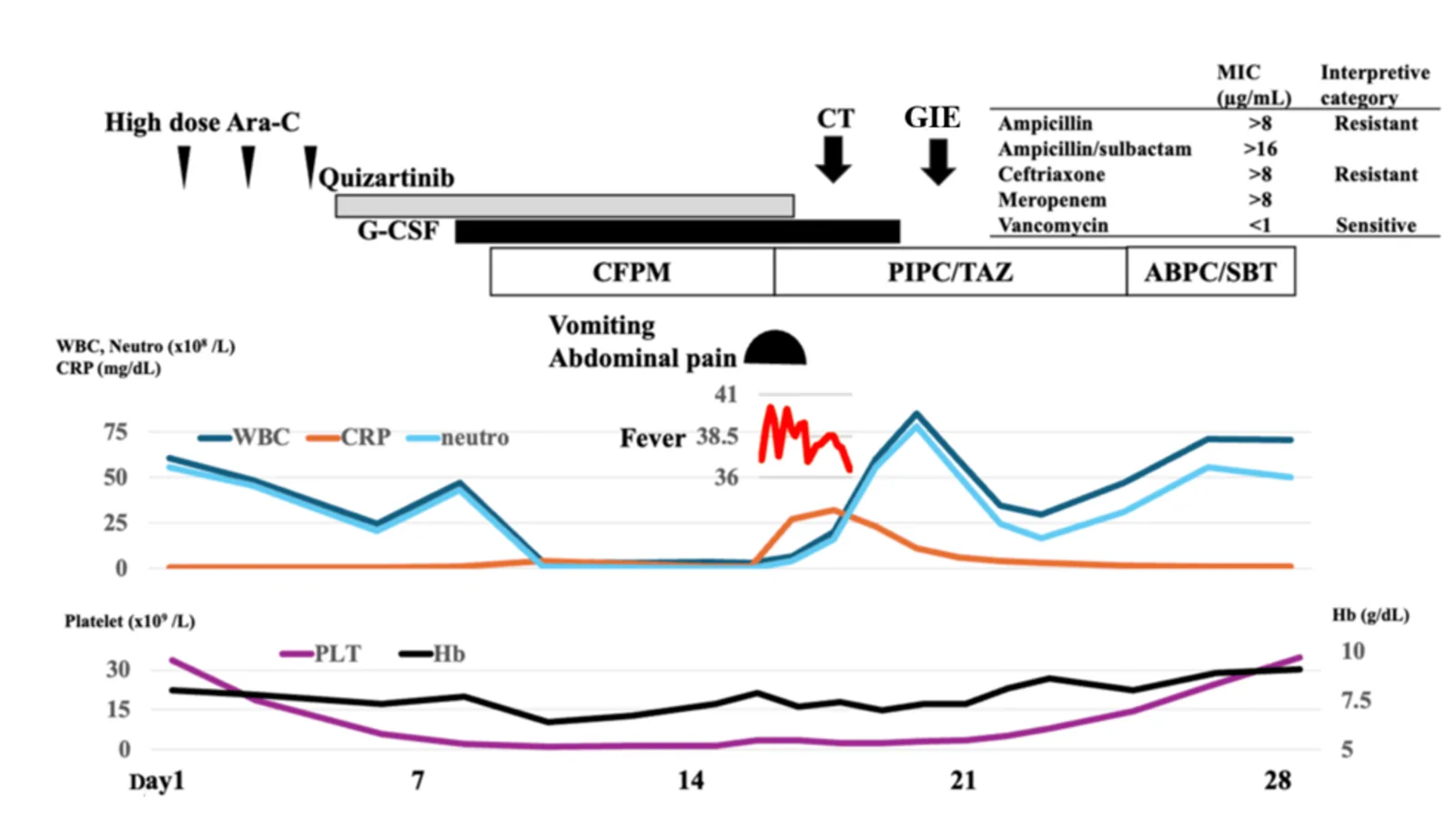
Patient’s clinical course. High-dose cytarabine was administered on days 1, 3, and 5. Quizartinib was administered from day 6 to day 17. G-CSF: granulocyte-stimulating factor; CFPM: cefepime; PIPC/TAZ: piperacillin/tazobactam; ABPC/SBT: ampicillin-sulbactam; WBC: white blood cell; CRP: C-reactive protein; Hb: hemoglobin; Plt: platelet; MIC: minimum inhibitory concentration; GIE: gastrointestinal endoscopy; CT: computed tomography. Figure 2 was created using Microsoft PowerPoint and Microsoft Excel, for Mac version 16 (Microsoft Corporation, Redmond, WA, USA).

After the endoscopic diagnosis on day 21, antimicrobial therapy was changed to ampicillin/sulbactam on day 21 and continued through day 32. Neutropenia persisted for eight days, with neutrophil recovery occurring on day 21. By day 28, follow-up CT demonstrated clear improvement in gastric wall thickening, and repeat upper gastrointestinal endoscopy on the same day showed marked resolution of the erosive lesions. The patient completed the antibiotics without recurrence of fever, gastrointestinal bleeding, or abdominal pain, and no surgical intervention was required (Figure 2).

Subsequently, the patient underwent allogeneic hematopoietic stem cell transplantation from an unrelated donor and achieved complete remission of AML. At the latest follow-up, the patient remained in hematologic complete remission with no recurrence of PG.

## Discussion

PG is a rare but life-threatening condition, with fewer than 500 cases reported in the literature [2]. The rarity of PG may reflect the stomach’s natural resistance to bacterial invasion, mediated by gastric acidity, an intact mucosal barrier, mucus production, rich vascularity, and local immune defenses. In our patient, chemotherapy-induced mucosal injury and profound neutropenia likely impaired these defenses, allowing *Enterococcus faecium* to invade the gastric mucosal tissue and cause PG. Here, we describe a patient with FLT3-ITD-positive AML who developed PG, diagnosed based on clinical, imaging, and pathologic features, during the neutropenic phase of consolidation chemotherapy with HiDAC plus quizartinib. Histopathological examination showed neutrophilic inflammation with Gram-positive bacterial aggregates, and culture of gastric biopsy specimens yielded *Enterococcus faecium*, a relatively rare pathogen associated with PG. The patient recovered following broad-spectrum antibiotic therapy, thereby enabling allogeneic hematopoietic stem cell transplantation.

Several factors likely contributed to the development of PG in this patient. Profound neutropenia, a hallmark of intensive consolidation chemotherapy, impairs host defense against bacterial invasion and is consistently identified as a major predisposing factor for hematologic malignancy-associated PG. HiDAC is well known to cause direct gastrointestinal mucosal injury, disrupting the epithelial barrier and facilitating transmural bacterial invasion. In addition, this patient received vonoprazan, a potassium-competitive acid blocker, which may increase gastric pH and alter the gastrointestinal microbiota [9]. The convergence of these factors, such as severe neutropenia, chemotherapy-induced mucosal injury, and altered gastric pH, created a highly susceptible host environment for the development of PG.

Although the literature on PG has gradually accumulated, PG occurring in patients with hematologic malignancies remains exceptionally uncommon. Previous cases have occurred in patients with acute leukemia in intensive chemotherapy, after transplantation, or, rarely, myeloid sarcoma [6-8,10,11]. These cases indicate that profound neutropenia, compromised host immunity, and chemotherapy-induced gastrointestinal mucosal injury increase susceptibility to bacterial invasion of the gastric wall.

Whether quizartinib-containing chemotherapy contributed to the development of PG remains uncertain. Upper gastrointestinal adverse events, predominantly characterized by mucosal injury, have been frequently reported with quizartinib monotherapy in clinical trials [12]. In the phase 3 QuANTUM-First trial, quizartinib combined with intensive chemotherapy improved survival in patients with newly diagnosed FLT3-ITD-positive AML, but myelosuppression was more frequently observed in the quizartinib arm [13]. The adverse events also showed slightly higher frequencies of upper abdominal pain, mucositis, and vomiting in the quizartinib-plus-chemotherapy group. While PG has not been established as a specific adverse event of quizartinib, the relationship cannot be inferred from a single case. This combination therapy may have indirectly increased susceptibility by prolonging neutropenia or exacerbating treatment-related mucosal injury.

The diagnosis of PG requires integration of radiologic, endoscopic, histopathologic, and microbiologic findings. Contrast-enhanced CT is particularly useful in the early phase and may show diffuse or segmental gastric wall thickening, perigastric fat stranding, and intramural low-density areas [5]. Endoscopic findings are nonspecific but may include erythematous, edematous, erosive, ulcerative, or necrotic mucosa. In this case, CT demonstrated gastric wall thickening, and endoscopy revealed diffuse edematous and erosive mucosal lesions. Histopathology showed inflammatory infiltrates with Gram-positive bacterial aggregates, and *Enterococcus* *faecium* was isolated from gastric biopsy specimens. These findings strongly supported the diagnosis of PG and helped exclude leukemic infiltration or other neoplastic involvement of the stomach.

*Streptococcus *species, especially *Streptococcus pyogenes*, have traditionally been considered the most common pathogens in PG [2]. Previous reports have also documented *Enterococcus*, *Staphylococcus*, Enterobacteriaceae, and polymicrobial infections, indicating substantial microbiologic heterogeneity. In hematologic malignancies, uncommon organisms such as *Stenotrophomonas maltophilia* and *Bacillus cereus *have also been reported [7,10]. This case is therefore notable in that *Enterococcus faecium* was isolated from a gastric biopsy culture, supporting the view that empirical antimicrobial therapy in neutropenic hosts should provide sufficiently broad coverage rather than focusing exclusively on streptococci. The patient’s clinical improvement coincided with neutrophil recovery on day 21, suggesting that restoration of host neutrophil function, rather than the specific antimicrobial regimen alone, may have played a major role in the clinical and radiologic resolution.

When perforation, extensive necrosis, or refractory septic deterioration is suspected, surgical treatment remains necessary in selected cases. In this case, the patient responded rapidly to antibiotic therapy after pathological and microbiological evaluation. Follow-up CT and endoscopy demonstrated marked resolution without the need for surgical procedures. The patient was managed conservatively despite severe presentation with shock and upper gastrointestinal bleeding, and resolution of PG was confirmed radiologically and endoscopically. Successful management of PG allowed subsequent unrelated allogeneic hematopoietic stem cell transplantation, and the patient has remained in complete remission without recurrence of PG, even during the severe immunosuppression after transplantation.

## Conclusions

We reported a case of a patient with FLT3-ITD-mutated AML who developed PG during the neutropenia phase of intensive consolidation therapy with HiDAC and FLT3 inhibitor quizartinib, in the setting of likely chemotherapy-associated mucosal injury. PG should be considered in neutropenic patients with hematologic malignancies who develop epigastric pain, fever, and upper gastrointestinal bleeding, particularly during intensive chemotherapy, including FLT3 inhibitor-containing therapy. Early contrast-enhanced CT evaluation, followed by endoscopy with biopsy and culture, and prompt broad-spectrum antibiotics are essential components of management. This case further shows that successful nonoperative management of PG can permit subsequent allogeneic hematopoietic stem cell transplantation.
